# Sustainable control of the bird cherry-oat aphid (*Rhopalosiphum padi* L.) in northwestern Ontario using *Beauveria bassiana* delivered by bumblebees

**DOI:** 10.3389/finsc.2025.1468262

**Published:** 2025-02-19

**Authors:** Jean Pierre Kapongo, Morel Libere Comlan Kotomale, Alphonsine Muzinga Bin Lubusu, Romuald Simo Nana, Donald Rostand Fopie Tokam, Grace Suzert Nottin Mboussou

**Affiliations:** ^1^ Applied Research Centre for Biodiversity (CRAB), Collège Boréal, Sudbury, ON, Canada; ^2^ Department of Agriculture, Collège Boréal, Sudbury, ON, Canada; ^3^ Research & Innovation Boréal (RIB), Sudbury, ON, Canada

**Keywords:** entomovectoring, *Beauveria bassiana*, biocontrol of aphids, bumblebees, natural enemies, bio-insecticide

## Abstract

*Rhopalosiphum padi* is one of the main vectors of barley yellow dwarf virus (BYDV), which affects the grain yield of oats. Several biological control strategies have been studied to control this pest, one of which is Bee Vectoring Technology (BVT) using bumblebees. We tested the efficacy of *Beauveria bassiana*, as vectored by bumblebees, and a direct spray application of neem (a natural bio-insecticide derived from the *Azadirachta indica* tree) on aphids. An assessment of the pest’s impact on the plots surveyed in northwestern Ontario revealed incidence rates of 80%. The use of bumblebees as a dispersal agent of *B. bassiana* significantly reduced the aphid population (0.542 ± 0.147b) compared to the untreated control (0.125 ± 0.069a). The application of diluted neem also showed a reduction in the aphid population (0.708 ± 0.221a). Although the products used controlled the pests, they had no effect on the aphid’s natural enemy, the ladybug. Therefore, the dissemination of *B. bassiana* by the bumblebee *Bombus impatiens* leads to a decrease in the vector *Rhopalosiphum padi* population and consequently reduces the severity of barley yellow dwarf disease in oat fields.

## Introduction

The oat (*Avena sativa* L.) is a species of cereal grain that is an important crop worldwide. FAO (1) estimates that 18.8 million metric tons of oats were produced in 2023, making it the sixth most widely grown cereal in the world. It is mainly used for animal feed, but also in the food industry for the preparation of certain bakery, pastry and breakfast products (2). According to Rasane et al. (3), oats are becoming increasingly popular due to their nutritional value in terms of dietary fiber and phytochemicals. In Canada, oats play a role in both providing a sustainable food supply for the population and driving the country’s economy (4). Canada is ranked as the second-largest oat producing country in the world (5). Total oat production in Canada was estimated at 2.6 million metric tons in 2023 (6). Unfortunately, the production of oats is subject to a number of biotic stresses, in particular insect pests, diseases and weeds, which affect grain yield, grain weight and percentage of groats, thus posing a threat to food security (7). Aphids are among the insect pests of oats, and the damage they cause is not limited to feeding on the plant’s phloem; they can also transmit viruses to the plant (8).

*Rhopalosiphum padi* (L.) is one of the aphids that transmits barley yellow dwarf virus to oats. This viral disease causes significant yield losses and economic damage of up to 3,790 kg/ha for every 1% increase in virus incidence (9). Originating in North America, this pest has spread to several regions of the world, including Europe, Asia, New Zealand, the United States and southern Canada (10). An assessment of the prevalence of barley dwarf yellow virus in winter wheat, carried out in southwestern Ontario, revealed the presence of *R. padi* in 50% of the fields tested (11). Although established in several regions of Ontario, there is little information on the incidence of this pest and the damage caused by the disease in northeastern Ontario. *R. padi* is typically managed using chemical insecticides. However, over the years, the pest has developed a resistance to these insecticides, limiting their usefulness (12, 13). In addition, intensive use of insecticides has profound repercussions on health, biodiversity and the environment. It can negatively influence the richness of natural enemies and agrosystems, thus weakening the management of pests by biological methods (14). Some cultural practices such as staggered planting dates, as well as the establishment of alternating strips and intercropping, have shown a reduction in the abundance of aphid populations in soybean fields (15). Few studies have been carried out on the economic impact of these different pest control practices. In addition, control methods using Bee Vectoring Technology (BVT) are still very poorly documented on oats. Natural control methods using entomopathogenic fungi and aqueous plant extracts could provide significant ways to sustainably manage *R. padi* in oat fields and reduce the environmental impact that comes with chemical pesticides. Several studies have evaluated the positive impact of BVT in the management of insect pests affecting crops such as barley, tomatoes, peppers and wheat (16–18). *Beauveria bassiana* (Ascomycota: Hypocreales) is an entomopathogenic fungus that produces toxic secondary metabolites, boosting its virulence in nearly 1,000 species of pathogenic insects (19). The fungus penetrates the insect cuticle and passes through the integument to the hemocoele, a nutrient-rich environment (20). The mycelium then transforms into a specialized yeast-like cellular phenotype, called hyphalic bodies or blastospores, to compromise the host’s defenses until the host is eliminated. Integrated pest management involving bumblebee vectoring of *B. bassiana* responds to the twin problems of labor management in agriculture and the exposure of sprayed products to natural enemies (21). The objective of this study is to evaluate the comparative effectiveness of the use of bumblebee entomovectoring of *B. bassiana* and the use of the bio-insecticide neem (*A. indica*) to manage *R. padi* in oat fields. The side effects of these products on natural enemies present in the environment, in particular ladybugs under natural conditions, was also evaluated.

## Materials and methods

### Experimental setting

**Figure 1:**
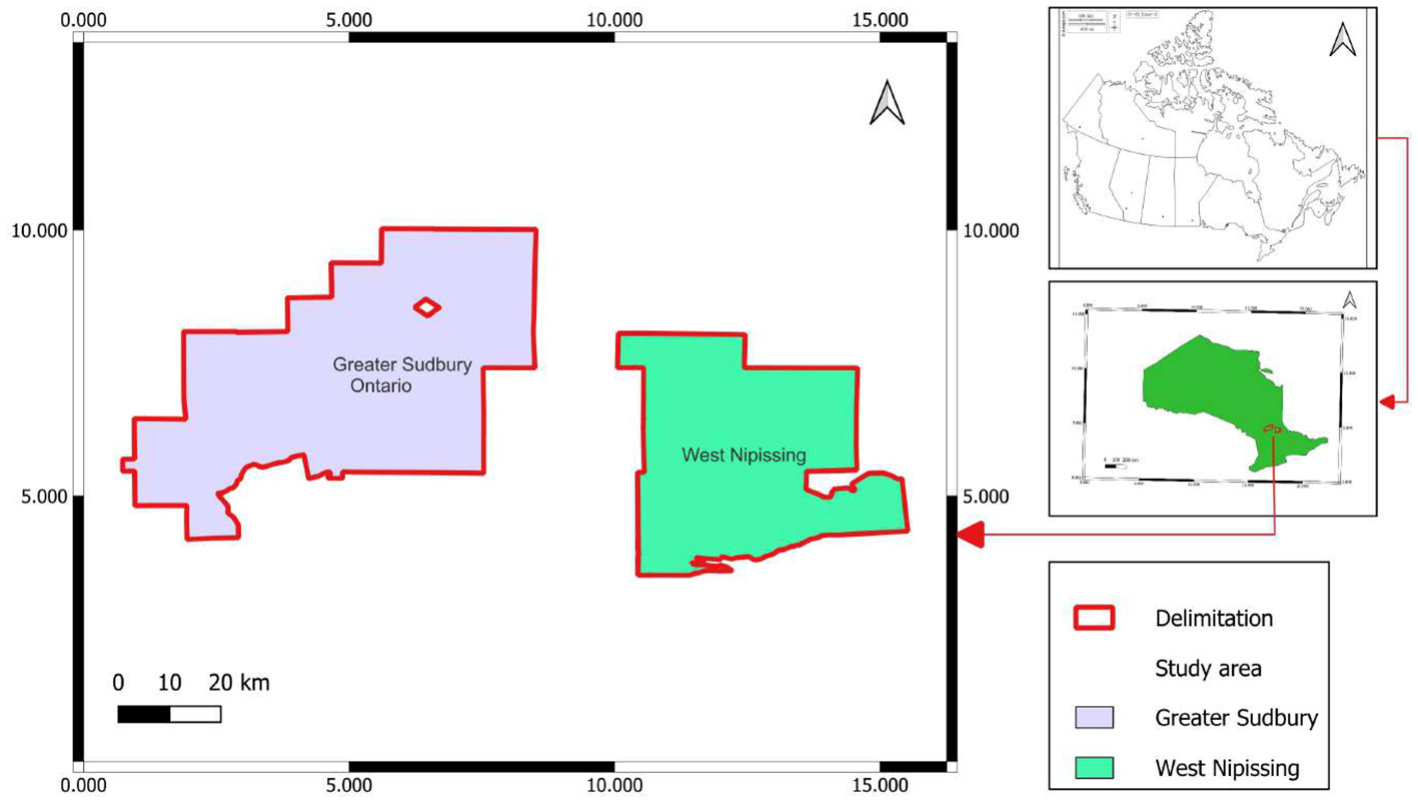
Geographic location of the study area.

Experiments were carried out at Leisure Farms, a family business that grows strawberries, raspberries, corn, fresh pumpkins, oats, etc., in the community of Sturgeon Falls, West Nipissing. From May to July 2023, weekly surveys were carried out to identify symptoms of barley yellow dwarf disease or the aphid *R. padi*, which transmits the barley yellow dwarf virus to oat plants. Six plots, identified by name (Solar, Quesnel A and B, Malette A and B, and Rheal 4A), were surveyed in the West Nipissing oat fields. Spores were cultivated and tested on infested insects at Collège Boréal’s Applied Research Centre for Biodiversity (CRAB) in Greater Sudbury (**Figure 1**).

To evaluate the entomovectoring potential of bumblebees, the commercial formulation of *B. bassiana*, strain GHA (BotaniGard^®^ 22WP, Lam International Corporation, Butte, MT, USA), supplied by Plant Products, was used at a concentration of 4.4 X 10^10^ conidia per kg of formula. Potato dextrose agar (PDA) culture medium was used to produce and propagate the fungus. The commercial formulation of Plantonix’s 100% cold-pressed neem oil was used, which is derived from pressing the fruits and seeds of the neem tree (*A. indica*). Bumblebee colonies (*Bombus impatiens*), supplied by Biobest Canada Ltd., Leamington, ON, Canada, were used as disseminators of *B. bassiana* in oat fields. Each colony was composed of a queen and 40 to 50 workers. The colonies were kept in commercial boxes provided by the manufacturer, 0.30 m x 0.23 m in size, that were equipped with an inoculum dispenser and a sugar solution (syrup) feeding system.

### Aphid population dynamics

To assess the dynamics of the aphid population, visual inspections were conducted with attention given to the various symptoms of barley yellow dwarf disease (stunted plants, yellowing of plants, etc.). Oat fields were scouted in a zigzag pattern to ensure coverage of the entire production area (22). The field was subdivided into subplots and ten oat plants were randomly observed in the first five rows over 10 m (distance). The number of plants observed and found to be diseased was recorded. A count of plants with one or more aphids was carried out to determine the density of the aphid population per plant, as well as a count of aphids to identify the insect’s different stages of development. To assess the severity of pest damage to oat plants, leaf damage was observed and scored using a scale from 0 to 3, where 0 = zero damage, 1 = 10% to 50% damage, 2 = 50% to 75% damage and 3 = dead plants (75% to 100%) (adapted from 23). After symptoms were noted, plant samples were collected for further observation and laboratory confirmation of aphid presence. Experiments were set up based on the current economic threshold of 12 to 15 aphids per tiller, determined by estimating the number of aphids present (24).

### Entomovectoring and biocontrol of aphids with *Beauveria bassiana*

The trials were set up on a plot that showed a high level of damage from aphids and barley yellow dwarf disease on oats. The viability of the commercial formulation of *B. bassiana* was evaluated prior to the start of the trials in order to calibrate the amount of product used. To do this, six 0.01 g samples of BotaniGard^®^ 22WP were mixed with 100 mL of distilled water and Tween 80 (0.1%). Then, 200 μL of the conidia suspension was added to 1 mL of PDA culture medium and incubated at 24 ± 1°C for 24 hours. After this period, four groups of 200 conidia were analyzed to determine their germination rate using a cell counting chamber (hemocytometer) with a microscope. The viability of the commercial formulation of *B. bassiana* was between 95% and 99% for all the trials conducted. The dispensers were filled with a spoonful (8 g) of formulation according to the designated treatment. The plants were placed under a mosquito net to avoid contamination from other plants and/or any bumblebees that escaped from the other hives.

### Preparation of neem extract concentration and application

The neem extract was prepared from the stock solution by adding a calculated amount of distilled water (v:v) and 1% of an added liquid detergent. Plants within the boundary of the micro-plot were sprayed with prepared neem extract. All emulsions were sprayed using a model H-7986 4-gallon, 15″ x 21″ backpack pressure hand sprayer.

### Treatments on the oat crop

Seven treatments were applied to the oat crop in the field (**Table 1**). They consisted of five specific formulations, most of which combined autoclaved maize flour, considered to be the most effective carrier, with BotaniGard^®^ 22WP (J. L. 25). The virulent effect of *B. bassiana* on the aphid population was evaluated by determining the average monitoring time for the insects exposed to the different concentrations (26). In addition, the mortality associated with the different preparations was evaluated on the same population by determining the lethal concentration likely to reduce 50% of the insect population (LC50). The dead insects were counted, collected and kept in a dark growth chamber at 25°C and 70% relative humidity for two weeks in order to promote fungal growth. Data on natural enemies were also recorded to assess the potential effect of the insecticide on natural enemies.

**Table 1:** Summary of products used and treatments carried out on *R. padi*.

Treatment	Commercial name	Combinations used
T1 (control): Untreated control	-	-
T2 (control): Bumblebees	Bee Vectoring Technology	25 g of maize flour
T3: Bumblebees (13.2 x 10^9^ conidia/g)	BotaniGard 22WP	3.3 x 10^10^ conidia/g + 25 g maize flour
T4: Bumblebees (4.4 x 10^9^ conidia/g)	BotaniGard 22WP	2.2 x 10^10^ conidia/g + 50 g maize flour
T5: Bumblebees (1.46 x 10^9^ conidia/g)	BotaniGard 22WP	1.1 x 10^10^ conidia/g + 75 g maize flour
T6: Spraying method	Plantonix’s commercial neem oil	14.19 mL neem oil + 1 liter water
T7: Spraying method	BotaniGard 22WP	3.3 x 10^10^ conidia/g + 1 liter water

### Statistical analysis

The data collected were analyzed using the XLSTAT software program, version 10.0. An analysis of variance (ANOVA) was carried out to compare variables based on classification: block, treatment, application (spraying of products) and yield. A Student-Newman-Keuls (SNK) test with a 5% threshold was then performed to compare the overall average rates of aphid leaf attack and the associated adult mortality rates.

## Results and discussion

### Aphid population density in the different plots surveyed

**Figure 2:**
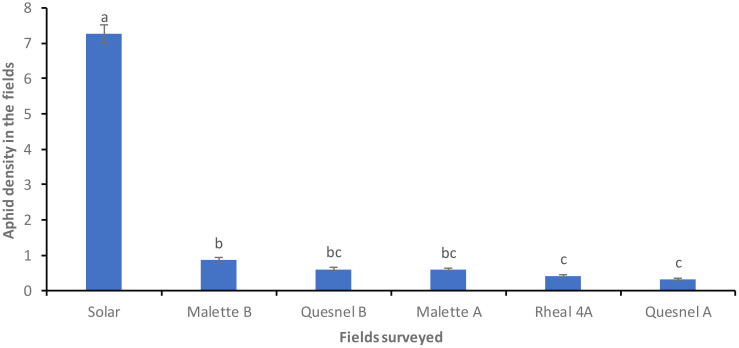
Average density of *R. padi* aphids per plant in the prospected fields before treatment. Averages with the same letter are not significantly different at P ≤ 0.05.

**Figure 3:**
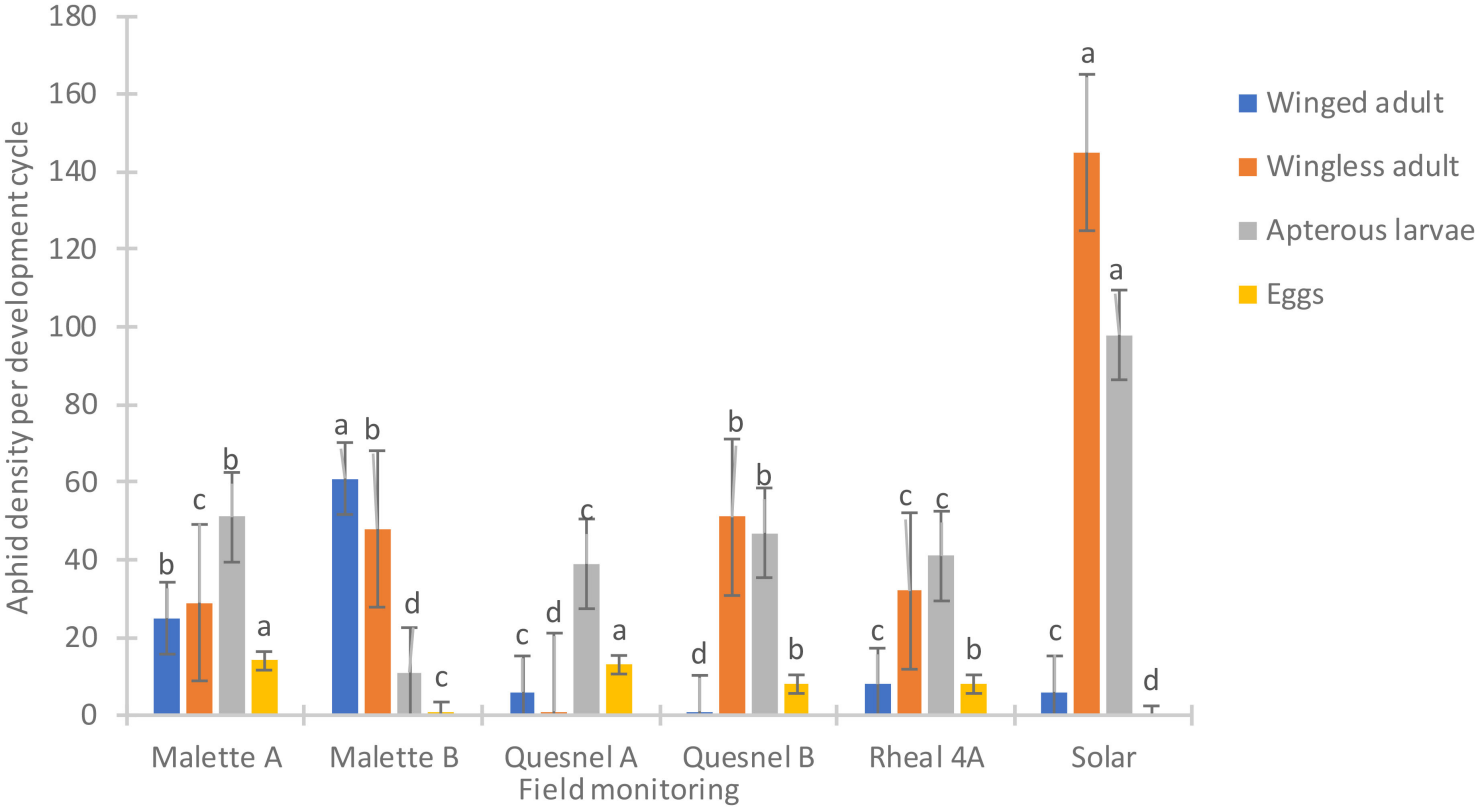
Distribution by development cycle of the average number of aphids on plots surveyed at Leisure Farm in 2023. Averages with the same letter are not significantly different at P ≤ 0.05.

**Figure 2** presents the population density of aphids (*R. padi*) based on the prospecting of the Leisure Farm plots (Solar, Malette A and B, Quesnel A and B, and Rheal 4A) for the period of May to July 2023. A significant difference was noted on the six plots surveyed (F = 550.85, Df = 5, P = 0.0001) (**Figure 2**). The average density of aphids per plant per plot was 0.31 ± 0.03c on Quesnel A (the least infested plot). Different stages of aphid development were noted (eggs, wingless larvae, wingless adults and winged adults). The average number of wingless larvae and adults was high on the Solar plot (51 larvae and 29 wingless adults) compared to the other plots (**Figure 3**).

The incidence and severity of aphids was 2 to 4 times higher on the Solar plot (80%) than on the Malette A and B plots, where it was 21.2% and 22.4%, respectively (**Tables 2**, **3**). These results confirmed the presence of the aphid in northeastern Ontario, a species previously reported in southwestern Ontario. This aphid has since spread to most parts of Ontario. The larval stage is the most damaging for plants because of the increased need for larvae to feed on plants in order to develop and grow. Britz (27) demonstrated, in a study on the relationship between the damage caused by *Spodoptera frugiperda* and yield loss in maize cultivation, that the degree of damage to plants was related to both the plant’s growth stage and the insect’s stage of development. Deole and Paul (28) explain that the larvae feed on the young leaves in sufficient enough quantities to eventually destroy them as the maize grows, which could explain the high presence of wingless larvae on the Solar plot. It should be noted that Solar was one of the last plots to be sown. A migration of adults from the formerly cultivated plots (Malette A and B, Quesnel A and B, and Rheal 4A) to the Solar plot for fresh young leaves resulted in a large population after egg laying. Jeanneau (29) reported a significant increase in aphid populations from June onwards, reaching a density peak between mid-July and early August, with a gradual decrease thereafter until the end of August. These observations support our results, as our surveys and experiments were conducted during the same period.

**Table 2:** Average aphid severity on surveyed plots.

Plot	Estimated average
Malette A	8.80 ± 2.53 e
Malette B	14.93 ± 6.20 b
Quesnel A	19.87 ± 4.56 c
Quesnel B	12.70 ± 3.42 d
Rheal 4A	14.13 ± 3.08 d
Solar	45.73 ± 13.72 a

Averages with the same letter are not significantly different at P ≤ 0.05.

**Table 3:** Impact of aphid damage on prospected plots.

Plots	Estimated average
Malette A	21.20 ± 6.62 e
Malette B	22.40 ± 7.33 d
Quesnel A	44.00 ± 9.44 b
Quesnel B	36.40 ± 9.30 b
Rheal 4A	41.60 ± 8.84 c
Solar	80.00 ± 20 a

Averages with the same letter are not significantly different at P ≤ 0.05.

### Aphid population density in the field to be treated

**Figure 4:**
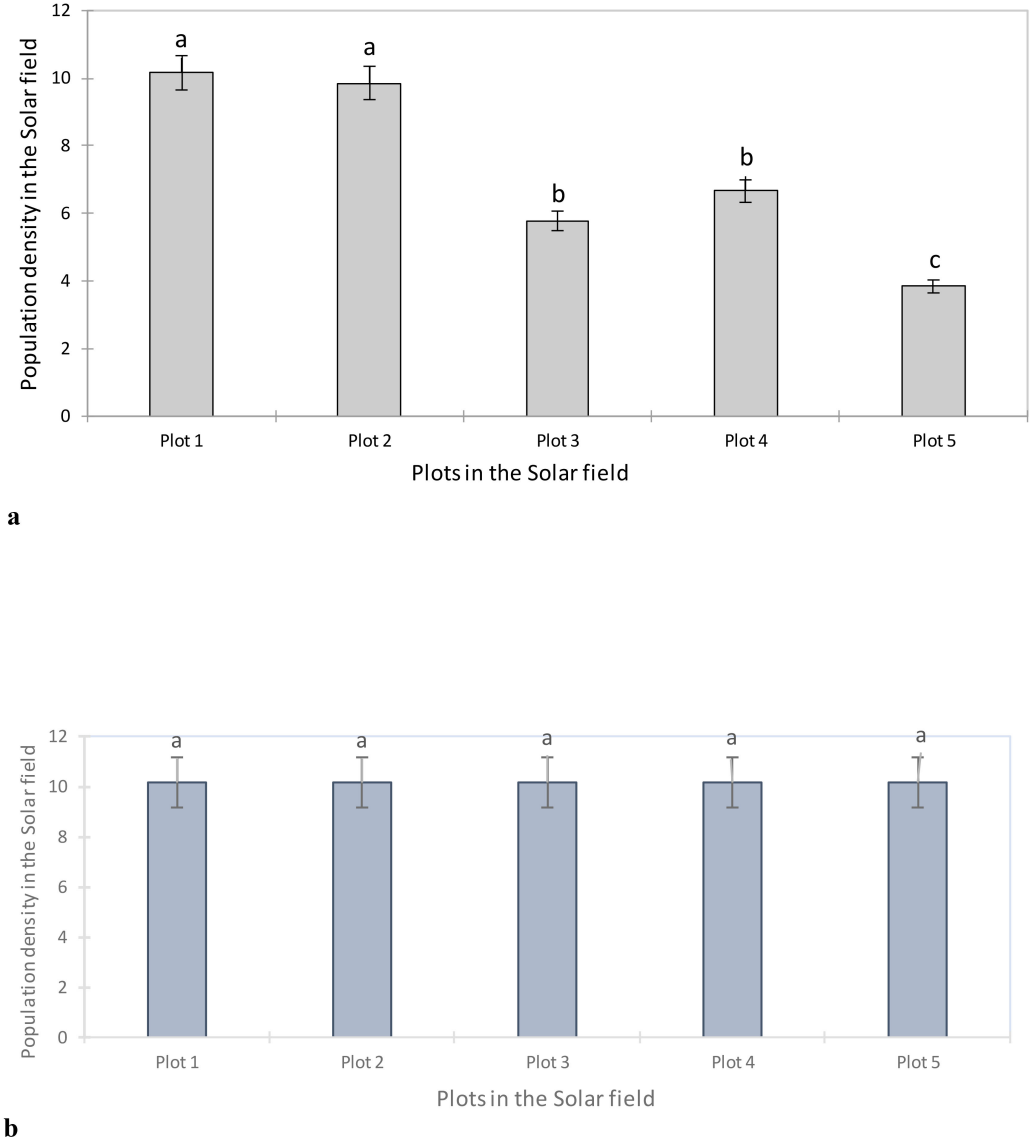
**(A)** Aphid density in the Solar field before artificial infestation. Averages with the same letter are not significantly different at P ≤ 0.05. **(B)** Aphid density after artificial infestation in the Solar field before treatment. Averages with the same letter are not significantly different at P ≤ 0.05.

**Figure 4A** shows the aphid density based on the different subplots sampled in the Solar field. Analysis of this figure shows a significant difference between aphid populations on the plots observed (F = 32.36; P < 0.0001). Plots 1 and 2 had similar aphid densities (10.16; 9.86) compared to plots 3, 4 and 5 (5.78; 6.66; 3.84).

To better assess the impact of the application of BVT with *B. bassiana* and neem, an artificial release of laboratory-raised aphids of the same age was carried out in the field. Field monitoring showed that all plots were infested with *R. padi* (**Figure 4B**).

### Effectiveness of different treatments on aphids and natural enemies

**Figure 5:**
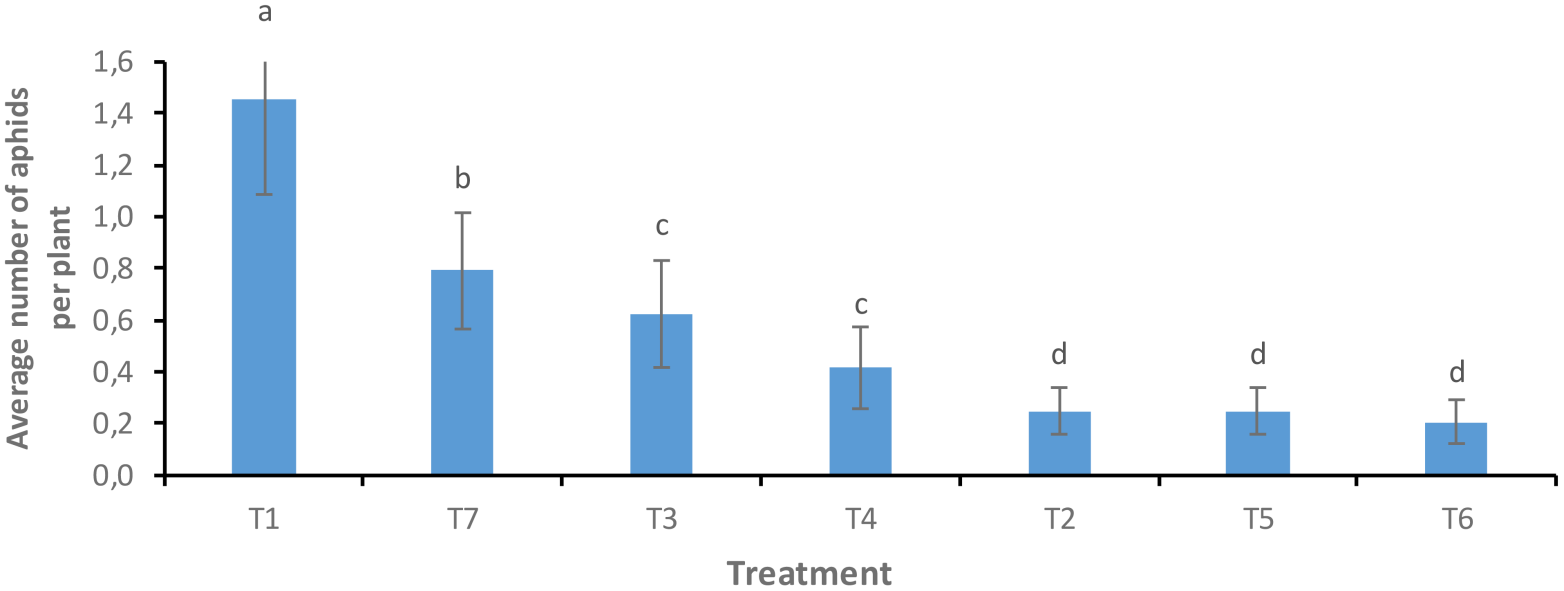
Average aphids observed per plant, by treatment. Averages with the same letter are not significantly different at P ≤ 0.05.

The Solar plot was selected as an experimental plot for the application of the various treatments because of its high aphid infestation rate. The dynamics of the aphid population were strongly influenced by the treatments on this plot. A significant difference was noted between the foliar spray treatments of neem oil and *B. bassiana* (T6 and T7) and the untreated control (T1), as well as between the treatments carried out by vectorization of bees with *B. bassiana* (T3, T4 and T5) and the control (T2) (F = 5.009; Df = 6; P = 0.0001). Lower aphid population density was noted with foliar spraying of neem oil (0.208 ± 0.08d) than with foliar spraying of *B. bassiana* (0.791 ± 0.33b) compared to the untreated control. Of the three treatments for bee vectorization with *B. bassiana* (T3, T4 and T5), only the T5 treatment showed significant control of the aphid population. These results suggest that the neem oil may have acted as a repellent for aphids. In addition, the bees were able to transmit the fungus *B. bassiana* to the fields, which would have caused a reduction in the aphid population, a result confirmed in the laboratory following the germination of the spores on the PDA medium (**Figure 5**).

**Figure 6:**
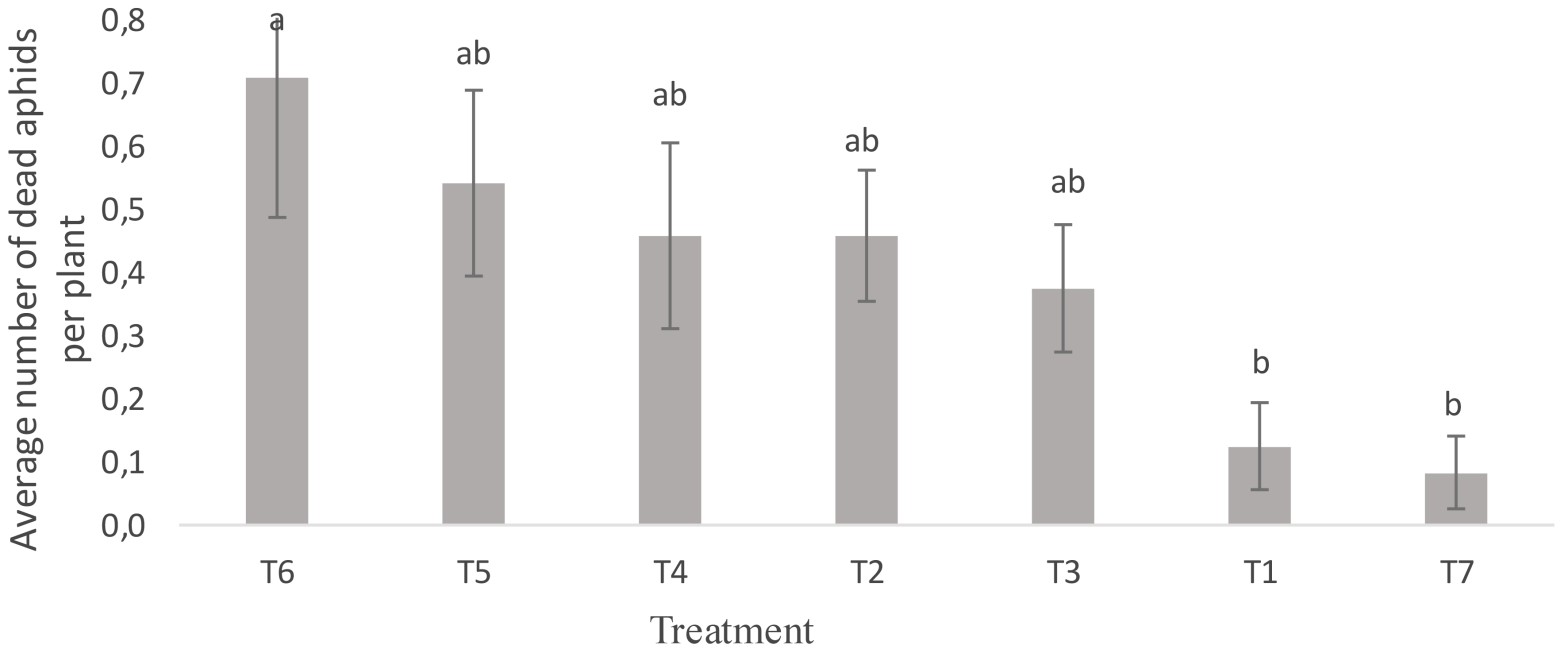
Average number of *R. padi* aphid deaths per plant, by treatment. Averages with the same letter are not significantly different at P ≤ 0.05. T1: Untreated control; T2: Control (Bumblebees + 25 g maize flour); T3: Bumblebees + 13.2 x 10^9^ conidia/g; T4: Bumblebees + 4.4 x 10^9^ conidia/g; T5: Bumblebees + 1.46 x 10^9^ conidia/g; T6: 14.19 mL neem oil + 1 liter water; T7: 3.3 x 10^10^ conidia/g + 1 liter water.

**Figure 6** shows the effect of treatments on the mortality of *R. padi* aphids. A significant difference was noted in the foliar spray treatments of neem oil and *B. bassiana* (T6 and T7) compared to the untreated control (T1) and in the bee vectorization treatments with *B. bassiana* (T3, T4 and T5) compared to the control (T2) (F = 2.72; P = 0.041). Foliar treatment with neem oil extracts (T6) resulted in a high aphid mortality of 0.71 ± 25.44%. Plants treated with bee vectoring (T5) also experienced high aphid mortality and sporulation rates (0.57 ± 20.66%). Foliar spraying of plants (T7), however, induced low aphid mortality rates (0.083± 2.98%). No significant differences were noted in factors such as time, severity and incidence of aphids after treatment, although the aphid population was reduced (P = 0.12; F = 1.94) (**Table 4**). These results show that both insecticide formulations provided aphid control. In addition, using BVT with 1.1 x 10^10^ conidia/g of *B. bassiana* disseminated the *B. bassiana* strain on the plants; this strain, which is infectious to aphids (30), then came into contact with the insects and caused their mortality. The infection of aphids by this fungus is very complex and depends on several factors, including the type of fungus used and the insect’s stage of development. The aphids collected on the Solar plot were mainly larvae and wingless adults. Our results corroborate those of Barbarin et al. (31), which revealed that short-term exposure to *B. bassiana* was highly virulent for insect pests, especially bedbugs. *B. bassiana* infects its insect host by using chitinase as a virulence factor (32). These enzymes digest the insect’s epidermis, allowing the mycelium to penetrate the cuticle, gain access to body fluids and destroy cells, resulting in the insect’s death.

**Table 4:** Effect of treatments on the average severity of R. padi aphids.

Treatment	Average
T1	2.20 ± 0.35a
T2	1.71 ± 0.32b
T3	1.21 ± 0.08b
T4	2.04 ± 0.40a
T5	1.50 ± 0.32a
T6	1.20 ± 0.16b
T7	1.45 ± 0.19b

Averages with the same letter are not significantly different at P ≤ 0.05.

Regarding aphid stages of development, Gujar et al. (33) found that younger larvae of *H. armigera* are more sensitive to different concentrations of *B. bassiana* than older larvae. Mehinto et al. (34) also showed that mortality rates of 30% to 100% could be noted on the larvae of *Maruca vitrata* (Lepidoptera: Crambidae) following inoculation with spores of *B. bassiana*. This entomopathogen can induce sufficient mortality in the various larval stages, in this case the first three developmental stages of the insect, when it is more vulnerable. Gao et al. (35)’s showed in their study of the immune responses of *Spodoptera frugiperda* to the pathogenicity of the PfBb strain of *B. bassiana* that the activity of protective enzymes and detoxifying enzymes was reduced in subjects in the first three stages. Also confirming our results, Elsharkawy et al. (36) demonstrated in their study the major role played by *B. bassiana* in suppressing young *R. ferrugineus* larvae as part of an integrated pest management program. These larvae had reduced enzymatic activity for 24 to 48 hours, which was much longer than in the control group. Bumblebees have previously contributed to the successful use of *B. bassiana* for aphid control. Mommaerts and Smagghe (37) have shown that bumblebees provide both plant pollination and entomovectorization in the fields. In addition, Shipp et al. (38) proved the efficacy of a treatment of 1.37 × 10^10^ conidia/g of *B. bassiana* inoculum as arthropod management for greenhouse tomatoes and peppers and consider BVT an approach that can be integrated into greenhouse insect control programs. *B. impatiens* has been identified as a true agent of *B. bassiana* and *Clonostachys rosea* for the control of *Botrytis cinerea* (16). This pollinator was found to be able to spread 46% and 57% of the spores on tomato flowers and leaves respectively, and 58.9% and 46.8% on pepper flowers and leaves respectively. By depositing the entomopathogen on the plant, the pollinator contributed to the control of *B. cinerea*.

The various treatments carried out do not have a significant effect on aphids’ natural enemies (for this study, ladybugs were used as an example) (**Table 5**). Ladybugs are well−known predators of aphids and other types of pests such as mites and mealybugs. The ladybugs were not infected with the entomopathogen due to their hard outer layer, which did not adhere to infection phases such as attachment, germination, and appressorium formation (39). The use of BVT with 1.1 x 10^10^ conidia/g considerably controlled the aphid population. Applying neem oil in the dose of 14.19 mL in 1 liter of water resulted in significant *R. padi* aphid mortality. Several previous studies have demonstrated the insecticidal effect of these biocontrol products. Camarda et al. (40) demonstrated that neem oil at a concentration of 20% was able to control up to 94.67% of the population of the mite *Dermanyssus gallinae* (Mesostigmata: Dermanyssidae). Azadirachtin, a compound contained in neem oil, is said to be the greatest contributor to mortality in wingless *R. padi* larvae. Schenk et al. (41) have shown that azadirachtin prevents the ecdysone activity that allowed the larvae to moult. Larvae prevented from moulting are unable to undergo metamorphosis and will eventually die.

**Table 5:** Effect of treatments on ladybugs (natural enemies of aphids).

Treatment	Average
T1	1.13 ± 1.13a
T2	0.79 ± 0.379a
T3	1.042 ± 1.04a
T4	1.125 ± 1.13a
T5	1.083 ± 1.08a
T6	0.917 ± 0.92a
T7	1.083 ± 1.08a

Averages with the same letter are not significantly different at P ≤ 0.05.

## Conclusion

This study was conducted to evaluate the potential of Bee Vectoring Technology using *Beauveria bassiana* and the application of aqueous neem extract to control the aphid *Rhopalosiphum padi* on oats. The results show the role of pollinators such as bumblebees in the transport of *B. bassiana*-based formulations on oat crops. The transport of spores of this fungus helped to significantly reduce the population of *R. padi* aphids. Similarly, the aqueous neem extract provides both aphid repellency and mortality. These two strategies can therefore be used as an alternative to chemical control to protect oats against *R. padi*. Note that neither of these insecticides had a significant effect on ladybugs, which confirms their safety and recommendation as sustainable control strategies for oat aphids. Bee Vectoring Technology is recommended as the strategy for oat growers to adopt over the direct application of neem oil using a sprayer, because BVT (especially T5: Bumblebee disseminating 1.46 x 10^9^ conidia/g of *B. bassiana*) not only reduces the aphid population, as neem did, but also pollinates the crop while protecting the aphid’s natural enemy (see **Table 5**: the ladybug population was the same in all treatments regardless of the use of *B. bassiana* and neem oil).

## Data Availability

The datasets presented in this study can be found in online repositories. The names of the repository/repositories and accession number(s) can be found in the article/supplementary material.
